# Spring viraemia of carp virus modulates p53 expression using two distinct mechanisms

**DOI:** 10.1371/journal.ppat.1007695

**Published:** 2019-03-29

**Authors:** Shun Li, Long-Feng Lu, Shu-Bo Liu, Can Zhang, Zhuo-Cong Li, Xiao-Yu Zhou, Yong-An Zhang

**Affiliations:** 1 Institute of Hydrobiology, Chinese Academy of Sciences, Wuhan, China; 2 Laboratory for Marine Biology and Biotechnology, Qingdao National Laboratory for Marine Science and Technology, Qingdao, China; 3 University of Chinese Academy of Sciences, Beijing, China; 4 State Key Laboratory of Agricultural Microbiology, College of Fisheries, Huazhong Agricultural University, Wuhan, China; University of Southern California, UNITED STATES

## Abstract

p53, which regulates cell-cycle arrest and apoptosis, is a crucial target for viruses to release cells from cell-cycle checkpoints or to protect cells from apoptosis for their own benefit. Viral evasion mechanisms of aquatic viruses remain mysterious. Here, we report the spring viremia of carp virus (SVCV) degrading and stabilizing p53 in the ubiquitin-proteasome pathway by the N and P proteins, respectively. Early in an SVCV infection, significant induction was observed in the S phase and p53 was decreased in the protein level. Further experiments demonstrated that p53 interacted with SVCV N protein and was degraded by suppressing the K63-linked ubiquitination. However, the increase of p53 was observed late in the infection and experiments suggested that p53 was bound to SVCV P protein and stabilized by enhancing the K63-linked ubiquitination. Finally, lysine residue 358 was the key site for p53 K63-linked ubiquitination by the N and P proteins. Thus, our findings suggest that fish p53 is modulated by SVCV N and P protein in two distinct mechanisms, which uncovers the strategy for the subversion of p53-mediated host innate immune responses by aquatic viruses.

## Author summary

Upon viral infiltration, host cells employ p53 to defend against infection. Thus, viruses need to inhibit these antiviral surveillance mechanisms in the host to efficiently spread to new hosts. To date, the evasion mechanisms against fish p53 remain unclear. In this study, we reveal that SVCV modulates host p53 expression by two distinct mechanisms. Through a series of experiments, we show that SVCV N protein bound and degraded host p53 through suppressing the K63-linked ubiquitination; SVCV P protein interacted with and stabilized p53 while enhancing the K63-linked ubiquitination; lysine residue 358 was the key site for p53 ubiquitination by the N and P proteins. Our findings shed light on the special evasion mechanisms of fish virus and expand our knowledge of the virus–host interactions that are responsible for regulating p53 in lower vertebrates.

## Introduction

The tumor suppressor p53 is a crucial cellular stress sensor that triggers apoptosis, cell-cycle arrest, and a series life biology processes by responding to environmental stresses such as DNA damage, hyperproliferative signals, and hypoxia [1, 2]. The corresponding cellular responses mediated by p53 depend on its transcriptional factor role to induce particular target genes [3, 4]. The activity of p53 demands tight limitations to the cell’s stabilization and the protein level of p53 is low in normal cells [5–7]. Previous studies have indicated that p53 participates in the defense against viral infection depending on its capacity to activate cell-cycle arrest or apoptosis via the transcription of target genes [8–10]. p53-dependent apoptosis has been identified as a powerful control to restrict virus infection, such as by limiting the infections of vesicular stomatitis virus (VSV), influenza A virus (IAV), herpes simplex virus (HSV), and poliovirus [11–16]. A putative explanation is that early apoptosis would be harmful to the virus as they should use the host’s resources for replication, thus impairing the production of newly formed viral particles [17].

However, viruses have evolved strategies to handle host p53 activity and thus facilitate viral replication and proliferation. Two pathways are invariably chosen by a virus for its own benefit: 1. Use p53 activity; p53 is employed by human cytomegalovirus (HCMV), respiratory syncytial virus (RSV), adenovirus, encephalomyocarditis virus (EMCV), and parainfluenza virus to promote viral replication [13, 18–20]. Moreover, p53 as a transcription factor transcripts the HCMV L44 protein required for virus replication, and 21 binding sites of p53 have been found in the virus genome [18]. 2. Counteract p53 activity. Kaposi’s sarcoma-associated herpesvirus (KSHV) ORF K8 interacts with p53 to inhibit its activity; the adenovirus E4-ORF6 protein degrades p53; HPV E7 suppresses p53 transcriptional activity; KSHV vIRF1 decreases p53 phosphorylation and promotes its ubiquitylation; the polyoma virus blocks the p53-mediated signaling pathway [21–24]. Thus, combat between the host’s innate immune response and viruses regarding p53 is complicated and pivotal, and although multiple correlative research studies have been accomplished in multiple species, this remains unclear for fish and fish virus.

Spring viremia of carp virus (SVCV) is an aquatic virus that belongs to the genus *Vesiculovirus* of the *Rhabdoviridae* family and causes remarkable mortality in common carp (*Cyprinus carpio*) [25]. It contains a ~11 kb negative-sense, single-stranded RNA and encodes five viral proteins in the following order: 3’-nucleoprotein (N protein), phosphoprotein (P protein), matrix protein (M protein), glycoprotein (G protein), and viral RNA-dependent RNA polymerase (L protein)-5’ [26]. The exact mechanisms of these viral proteins functioning on SVCV replication and proliferation are poorly known. According to the studies that examined rhabdovirus, N protein interacts with viral RNA to construct a nucleocapsid for assembly, M protein is involved in the budding formation, phosphorylated P protein associates with L protein to form an activated viral polymerase that interacts with the RNA template, and G protein participates in viral endocytosis [27].

Previous studies by our lab have reported the strategies of SVCV to evade the host IFN response. As a decoy of TBK1, SVCV P protein leads to decreased IRF3 phosphorylation and the N protein degrades MAVS via the K48-ubiquitin-proteasome pathway; thus, both these proteins significantly reduce the host IFN transcription to facilitate viral proliferation [28, 29]. We further explore the strategies of aquatic virus innate immune evasion pathways here by reporting fish p53 as an identical target that is modulated by SVCV N and P proteins to respectively decrease and stabilize with decreasing and increasing K63-linked ubiquitination at the K358 site. These findings shed light on the mechanisms of p53 regulated by aquatic viruses in lower vertebrates.

## Results

### SVCV infection renders host cell S phase arrest

**Fig 1:**
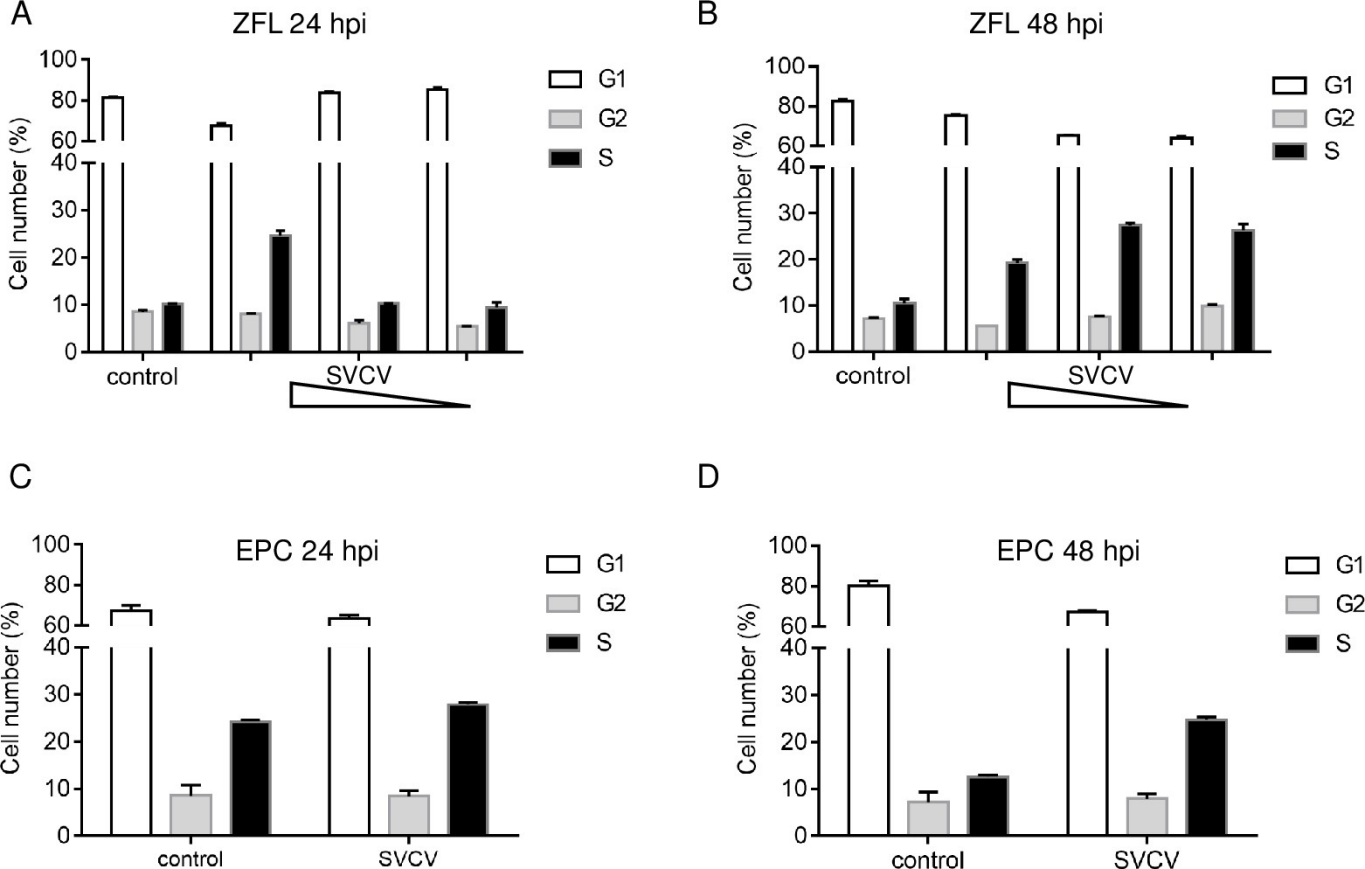
Cell cycle distribution of SVCV-infected cells. **(A and B)** Flow cytometry analysis of cell cycle profiles in ZFL cells with control or SVCV infection (MOI = 0.1, or 1, or 10) for 24 h (A) and 48 h (B). Cells were fixed in ethanol and stained with PI. The percentages of each cell cycle phase (G1, G2, S) were output and analyzed by ModFit LT software. **(C and D)** Cell cycle profiles in EPC cells were measured after SVCV (MOI = 10) infected for 24 h (C) and 48 h (D). The data are mean ± SD of three independent experiments. *p < 0.05, versus control infection in the same kinds of cells at same time points.

Viral infection invariably leads to a changed cellular statement, including cell-cycle arrest and apoptosis [1]. The effect of stimulating fish cells with fish virus remains unclear. We identified the influence of SVCV infection on fish cell-cycle progression by infecting ZFL cells from the liver of zebrafish with different doses and times as indicated and we analyzed the cell-cycle progression by flow cytometry. At 24 hpi, the cell population of the S phase of SVCV-infected cells in (MOI = 10) was close to 2.3-fold higher than that among mock-infected cells, and the groups of MOI = 1 and MOI = 0.1 were obscure (Fig 1A). At 48 hpi, the S phase accumulations of the three infection groups were respectively increased by 1.8-, 2.3-, and 2.2-fold compared to the mock infection cells (Fig 1B). EPC cells are the usual infection cell line model for SVCV and are effective at SVCV infection. At 24 hpi, there is no obvious difference in S phase accumulation between the mock and infected cells, while SVCV infection triggers a 1.8-fold cell-cycle progression that delays the S phase at 48 hpi (Fig 1C and 1D). These results demonstrated that SVCV leads fish cells to a remarkable S phase accumulation.

### The p53 protein level was decreased by SVCV N protein

**Fig 2:**
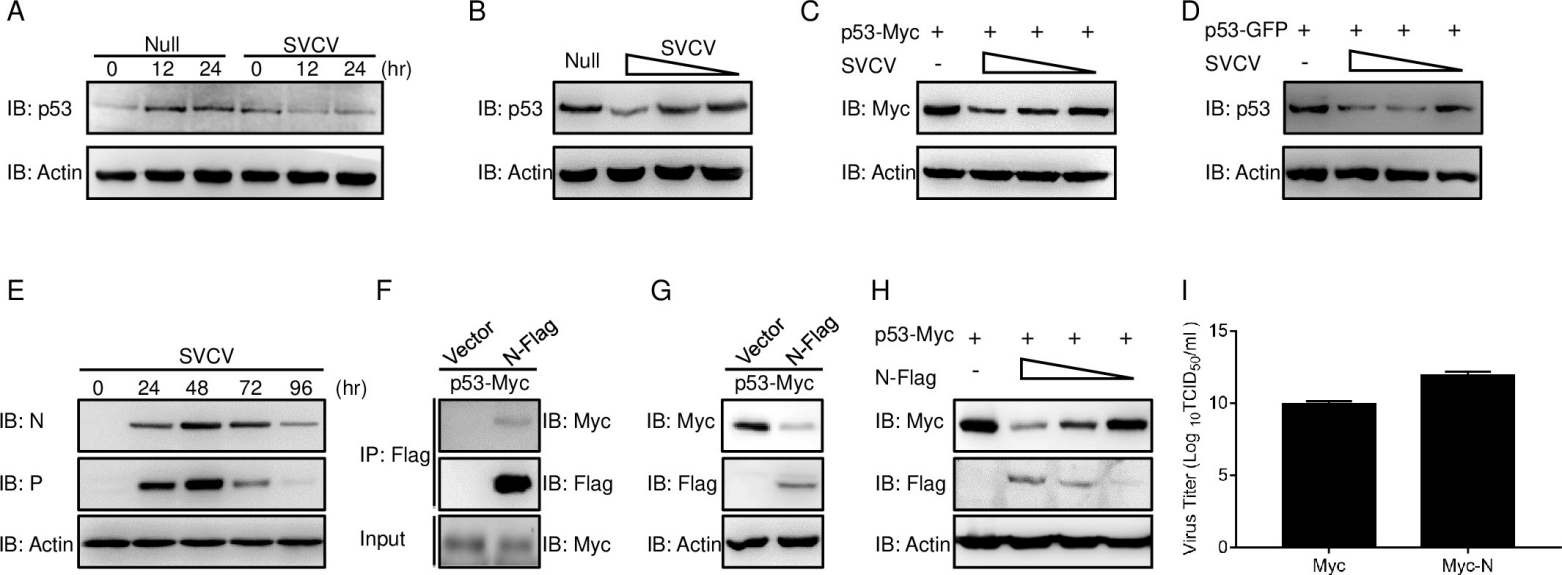
N protein of SVCV inhibits p53 expression. **(A)** SVCV infection decreases the expression of endogenous p53 protein. ZFL cells were seeded in 6-well plates and uninfected (null) or infected with SVCV (MOI = 1) at various time points, and the whole-cell lysates (WCL) were detected by IB, with the anti-p53 and anti-β-actin Abs. **(B)** High dose of SVCV infection attenuates the expression of endogenous p53 protein. ZFL cells were seeded in 6-well plates and infected with SVCV at the dose (MOI = 10, or 1, or 0.1) for 24 h prior to being harvested for IB analysis of WCL with the Abs indicated. **(C)** SVCV infection inhibits exogenous p53 expression in a dose-dependent manner. EPC cells were seeded in 6-well plates and transfected with 2 μg p53-Myc for 24 h, then cells were infected with SVCV (MOI = 10, or 1, or 0.1) for 24 h prior to being harvested for IB analysis of WCL with the anti-Myc and anti-β-actin Abs. **(D)** p53 is downregulated by SVCV in the stably expressed cell line. p53-stably expressed ZFL cells were seeded in 6-well plates and infected with SVCV (MOI = 10, or 1, or 0.1) for 24 h, then harvested for IB analysis with the anti-p53 and anti-β-actin Abs. **(E)** The expression of SVCV N and P protein at protein level. ZFL cells were seeded in 6-well plates and uninfected (null) or infected with SVCV (MOI = 0.1) at various time points, and the WCL were detected by IB, with the anti-N protein, anti-P protein and anti-β-actin Abs. **(F)** p53 associates with N protein in a mammalian overexpression system. HEK 293T cells seeded in 10 cm^2^ dishes were transfected with the indicated plasmids (5 μg each). After 24 h, cell lysates were IP with anti-Flag-affinity gels. Then the immunoprecipitates and WCL were analyzed by IB with the Abs indicated. **(G)** Overexpression of N protein declines the expression of p53. EPC cells were seeded in 6-well plates and transfected with 1 μg p53-Myc together with1 μg empty vector, or N-Flag for 24 h. Then the WCL were subjected to IB with the Abs indicated. **(H)** N protein blocks the expression of p53 in a dose-dependent manner. EPC cells were seeded in 6-well plates and transfected with 1 μg p53-Myc and 1 μg empty vector, or N-Flag (1, 0.5, or 0.25 μg) for 24 h. Then the WCL were subjected to IB with the Abs indicated. **(I)** Increase of virus replication by overexpression of the N protein. EPC cells seeded in 24-well plates overnight were transfected with 0.5 μg N-Myc or empty vector. At 24 h post-transfection, cells were infected with SVCV (MOI = 10) for 48 h, the culture supernatants were collected and then the viral titer was measured by standard plaque assays. Error bars are the SDs obtained by measuring each sample in triplicate. Asterisks indicate significant differences from control (*, *p* < 0.05). All experiments were repeated for at least three times with similar results.

A series of studies have suggested that p53 expression interference is a pivotal mechanism for multiple viruses to force host cells to enter their replicative S phase, favoring virus replication [30]. Since the above study means that the significantly increased fish cell population in the S phase is infected by SVCV, the expression of p53 needs to be clarified. In mock-infected cells, p53 was increased from 12 hpi to 24 hpi to present a stable expression, while this was decreased significantly in SVCV-infected cells at 12 hpi and 24 hpi (Fig 2A). Then, stimulated with a dose-dependent infection of SVCV, p53 expression was inhibited under high-dose viral infection and unchanged in the low-dose group (Fig 2B). We illustrated whether the decline of p53 was caused by mRNA or protein suppression by cloning the ORF of p53 into the eukaryotic expression vector with a Myc tag. Upon transfection with p53-Myc and infection with SVCV, the expression of p53 was attenuated dose-dependently (Fig 2C). Then, the p53-stably expressed ZFL cell line was established and infected with SVCV, the result was consistent with the above (Fig 2D). These results demonstrated that SVCV interferes with the host p53 protein level expression. As p53 is blunted by SVCV infection, the regulation relationship between p53 and SVCV proteins should be identified. First, the expression level of viral protein was monitored. The N and P proteins were expressed at 24–96 hpi (Fig 2E). Subsequently, the co-overexpression of SVCV N protein with Flag tag (N-Flag) and p53-Myc, the anti-Flag antibody (Ab)-immunoprecipitated protein complexes containing N protein were recognized by the anti-Myc Ab, meaning that N protein is associated with p53 (Fig 2F). Therefore, according to the above data, the N protein of SVCV is associated with p53. Based on that result, the SVCV N protein interacts with p53 and the effect of p53 expression or stability can be examined. Upon co-transfection of p53-Myc and N-Flag, the cell lysate was checked by immunoblot (IB) with specified Abs. Compared with the control group, overexpression of the N protein had obviously declined p53 expression (Fig 2G). Consistent with these observations, we focus on how functional N protein had decreased the p53 expression. Upon overexpression with different doses of N protein, the result from IB showed that p53 was abrogated dose-dependently (Fig 2H). Subsequent infection with SVCV revealed an up to about 180-fold increase of virus titer in N protein-transfected cells (Fig 2I). A previous study demonstrated that the N protein also restricted IFN expression; therefore, the enhanced virus proliferation should be mediated by the multifunction of the N protein. These data demonstrated that SVCV N protein subverted the host’s p53 protein expression.

### p53 was attenuated by the N protein during the decreased K63-linked ubiquitination

**Fig 3:**
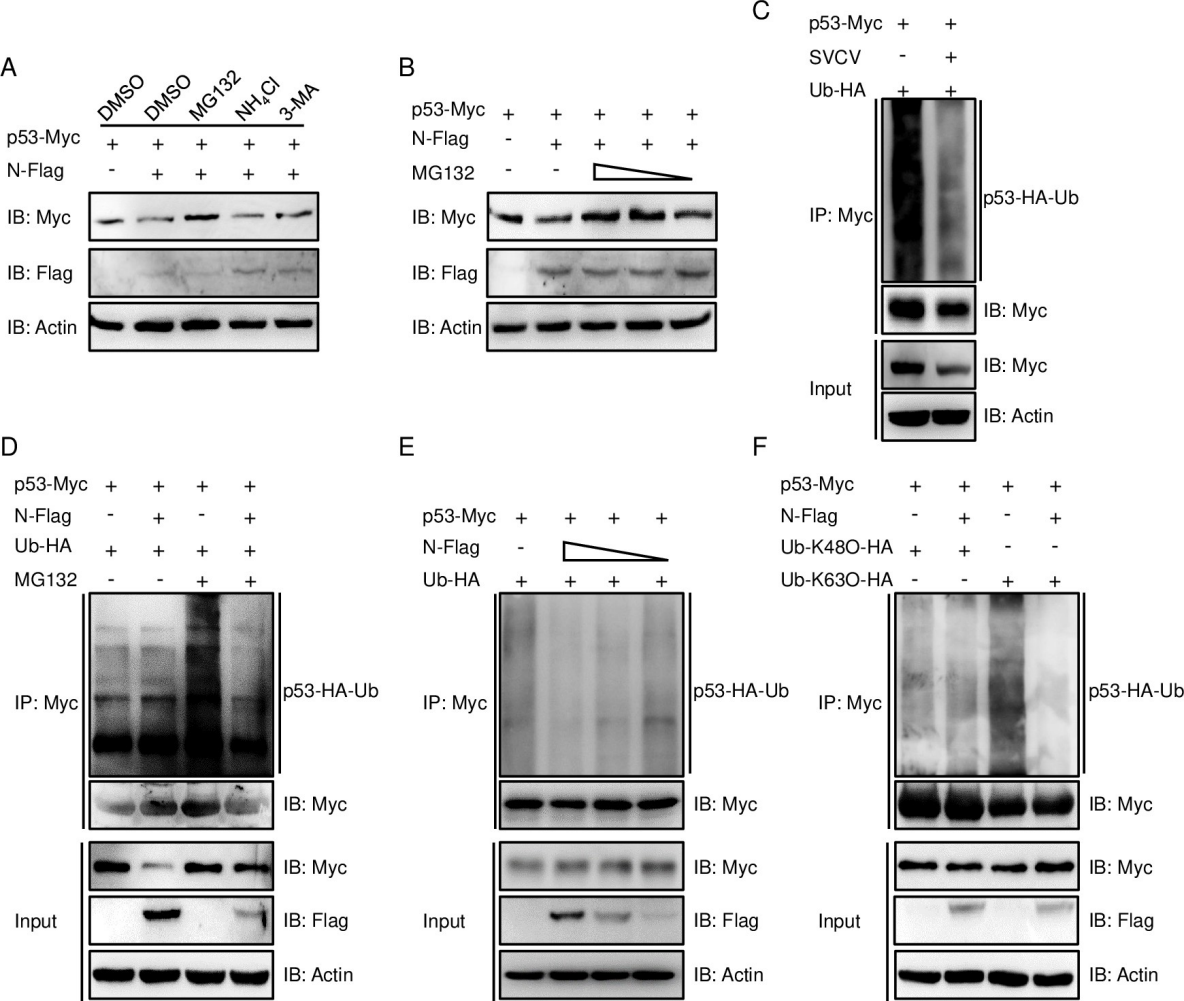
N protein of SVCV decreases K48-linked ubiquitination of p53. **(A)** Effects of inhibitors on N protein-mediated destabilization of p53. EPC cells were seeded in 6-well plates overnight and transfected with 1 μg p53-Myc and 1 μg empty vector, or N-Flag. At 18 h post-transfection, the cells were treated with the indicated inhibitors for 6 h prior to being harvested for IB analysis of WCL with the anti-Myc, anti-Flag, and anti-β-actin Abs. **(B)** The N protein-induced p53 declination was rescued by MG132 in a dose-dependent manner. EPC cells were seeded in 6-well plates overnight and transfected with 1 μg p53-Myc and 1 μg N-Flag or empty vector. At 18 h post-transfection, the cells were treated with DMSO or MG132 (10, 20, or 40 μM) for 6 h. Then the WCL were subjected to IB with the Abs indicated. **(C)** SVCV infection suppresses the ubiquitination of p53. EPC cells were seeded in 10 cm^2^ plates and transfected with 5 μg p53-Myc and 1 μg HA-Ub. At 24 h post-transfection, the cells were uninfected or infected with SVCV (MOI = 10) for 18 h, then the cells were treated with MG132 for 6 h. Cell lysates were IP with anti-Myc-affinity gels. Then the immunoprecipitates and WCL were analyzed by IB with the Abs indicated. **(D)** N protein decreases the ubiquitination of p53. EPC cells were seeded in 10 cm^2^ dishes and transfected with 5 μg p53-Myc, 5 μg N-Flag or empty vector, and 1 μg HA-Ub. At 18 h post-transfection, the cells were treated with DMSO or MG132 for 6 h. Cell lysates were IP with anti-Myc-affinity gels. Then the immunoprecipitates and WCL were analyzed by IB with the Abs indicated. **(E)** N protein negatively regulates the ubiquitination of p53 in a dose-dependent manner. EPC cells were seeded in 10 cm^2^ dishes and transfected with 5 μg p53-Myc, 5 μg empty vector, or N-Flag (5, 2.5, or 1 μg), and 1 μg HA-Ub. At 18 h post-transfection, the cells were treated with MG132 for 6 h. Cell lysates were IP with anti-Myc-affinity gels. Then the immunoprecipitates and WCL were analyzed by IB with the Abs indicated. **(F)** N protein decreases K63-linked ubiquitination of p53. EPC cells were seeded in 10 cm^2^ dishes and transfected with 5 μg p53-Myc, 5 μg N-Flag or empty vector, and 1 μg Ub-K48O-HA or Ub-K63O-HA. At 18 h post-transfection, the cells were treated with MG132 for 6 h. Cell lysates were IP with anti-Myc-affinity gels. Then the immunoprecipitates and WCL were analyzed by IB with the Abs. All experiments were repeated for at least three times with similar results.

In the above results, the protein level expression of p53-Myc, which contained a strong promoter (CMV promoter), was disrupted by N protein; therefore, we speculate that N protein could regulate p53 at the protein level. N protein and p53 were overexpressed and the cells were then treated with one of three agents: MG132 (an inhibitor for the proteasome pathway), NH_4_Cl (a lysosomal inhibitor), or 3-MA (an inhibitor for the autophagosome pathway), with DMSO treatment as the control group. In addition to the above result, the expression of p53 protein co-expressed with N protein was weaker than its co-expression with the empty vector, and such attenuation was significantly rescued by MG132 (Fig 3A). Subsequently, the rescue experiment was performed with different doses of MG132 and the declination of p53 regulated by N protein was rescued by MG132 dose-dependently (Fig 3B). These observations indicated that p53 was negatively regulated by N protein in Ub-proteasomal degradation. We further clarified whether p53 was decreased in the Ub-proteasome pathway by co-transfecting p53-Myc and Ub-HA and then infecting with SVCV, then immunoprecipitating p53-Myc; IB result demonstrated that SVCV suppressed the ubiquitination of p53 (Fig 3C). Subsequently, p53-Myc, N-Flag, and Ub-HA were co-transfected in either the presence or absence of MG132. The result of IB demonstrated that the N protein suppressed the ubiquitination of p53 (Fig 3D). Following the expression of N protein in several doses, the ubiquitination of p53 presented in a dose-dependent manner (Fig 3E). K48 and K63, which are lysines at positions 48 and 63 of ubiquitin and linked with polyubiquitin chains, represent the two canonical polyubiquitin chain linkages. Many studies have suggested that while target proteins were degraded by K48-linked polyubiquitin chains in a proteasome-dependent manner, they were stabilized by K63-linked polyubiquitin chains. Herein, whether the attenuation of p53 was mediated by the increase of K48-ubiquitination or the decrease of K63-ubiquitination should be identified. Upon co-transfection with p53, N protein, Ub-K48O, or Ub-K63O with corresponding tags, the K48-linked ubiquitination of p53 was unchanged under N protein regulation; however, the K63-linked ubiquitination of p53 was significantly impaired in the same condition (Fig 3F). As noted above, the observation suggested that p53 was negatively regulated by SVCV N protein via the attenuation of K63-ubiquitination.

### The mRNA level of p53 was declined by SVCV N protein

**Fig 4:**
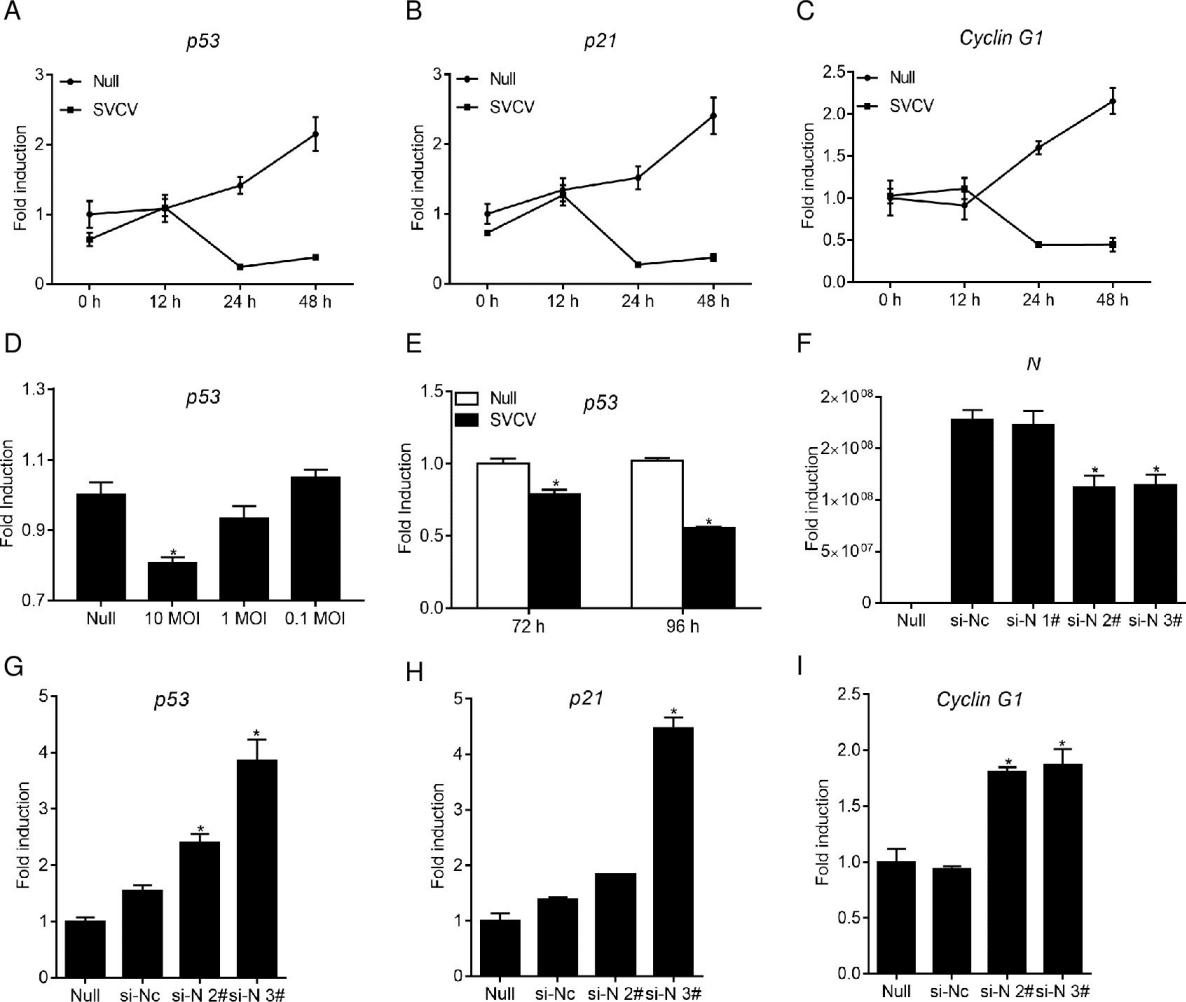
N protein of SVCV inhibits the p53 transcription. **(A-C)** qPCR detection of the transcriptional levels of *p53*, *p21*, and *cyclin G1* under SVCV infection. ZFL cells were seeded on 6-well plates overnight and infected with SVCV (MOI = 10). At the time points 12, 24, and 48 h, total RNA was extracted for further qPCR assays. **(D)** The expression of p53 under SVCV infection. ZFL cells were seeded in 6-well plates and uninfected (null) or infected with SVCV (MOI = 10, or 1, or 0.1) for 24 h, total RNA was extracted for further qPCR assays. **(E)** The expression of p53 at late stage of viral infection. ZFL cells were seeded in 6-well plates and uninfected (null) or infected with SVCV (MOI = 0.1) at various time points, total RNA was extracted for further qPCR assays. **(F)** Effects of RNAi on expression of SVCV N. EPC cells were seeded on 6-well plates overnight and transfected with 100 nM si-N#1, si-N#2, si-N#3, or si-NC. At 6 h post-transfection, the cells were infected with SVCV (MOI = 10). At 24 h post-infection, total RNAs were extracted to examine the transcriptional levels of *n*. **(G-I)** Effects of RNAi on SVCV-induced *p53*, *p21* and *cyclin G1* transcription. EPC cells were seeded in 6-well plates and transfected with 100 nM si-NC, si-N#2 or si-N#3. At 6 h post-transfection, cells were uninfected or infected with SVCV (MOI = 10) for 24 h before qPCR analysis was performed. The relative transcription levels were normalized to the transcription level of the *β-actin* and represented as fold induction relative to the transcription level in control cells, which was set to 1. Error bars represent SDs obtained by measuring each sample in triplicate. Asterisks indicate significant differences from the control (*, *p* < 0.05).

Meanwhile, in stimulation with SVCV for indicated time points, the RNA of ZFL cells were employed to examine the transcripts. At 12 hpi, the *p53* mRNA expressions were nearly equal between the infected and mock-infected groups, whereas it was swiftly down-regulated, and lower by about 5.2-fold and 7.3-fold compared to the control group (Fig 4A). As it is a key transcript factor, several downstream genes such as *p21* and *cyclin G1* responded to *p53*. Therefore, the mRNA abundance of *p21* and *cyclin G1* were also detected. In addition to the expression pattern of *p53*, the *p21* transcripts amount in SVCV-infected cells and mock-infected cells were similar at 12 hpi and lower in the infected group by about 4.6-fold and 6.1-fold compared to the mock group at 24 hpi and 48 hpi, respectively (Fig 4B). Furthermore, the expression of *cyclin G1* was lower by 3.5-fold and 4.8-fold than the control group when infected with SVCV at 24 hpi and 48 hpi (Fig 4C). Subsequently, the result of infection with different doses of SVCV demonstrated that the p53 mRNA level was blunted by SVCV dose-dependently (Fig 4D). Then, the expression of p53 at 72 hpi and 96 hpi of SVCV infection was also assayed, and found to be lower than that in the control groups (Fig 4E). These data suggest that the mRNA of p53 and related downstream genes were down-regulated in SVCV infection.

Subsequently, the mRNA level of p53 regulated by N protein was also monitored. There, small interfere RNA (siRNA) (si-N 1#, si-N 2#, and si-N 3#) that were designed for N protein were transfected into EPC cells and were then infected with SVCV. In contrast with the control group, the groups of si-N 2# and si-N 3# interfered with the SVCV *n* gene expression efficiently (Fig 4F). si-N 2# and si-N 3# were chosen for the following assessment. The cells’ overexpression of si-N 2# or si-N 3# and infection with SVCV in real-time PCR assay manifested that *p53* was strikingly upregulated when the expression of the SVCV *n* gene was interfered (Fig 4G). Next, the expression of *p21* and *cyclin G1* under the disruption of N protein during SVCV infection was also confirmed. Similar to the result for *p53*, remarkable increases of *p21* and *cyclin G1* expression at the mRNA level were observed in the siRNA group (Fig 4H and 4I). Overall, these results indicate that SVCV N protein negatively regulated p53 expression at both the protein and mRNA level.

### p53 was upregulated by the P protein via the increased K63-linked ubiquitination in the late stage of SVCV infection

**Fig 5:**
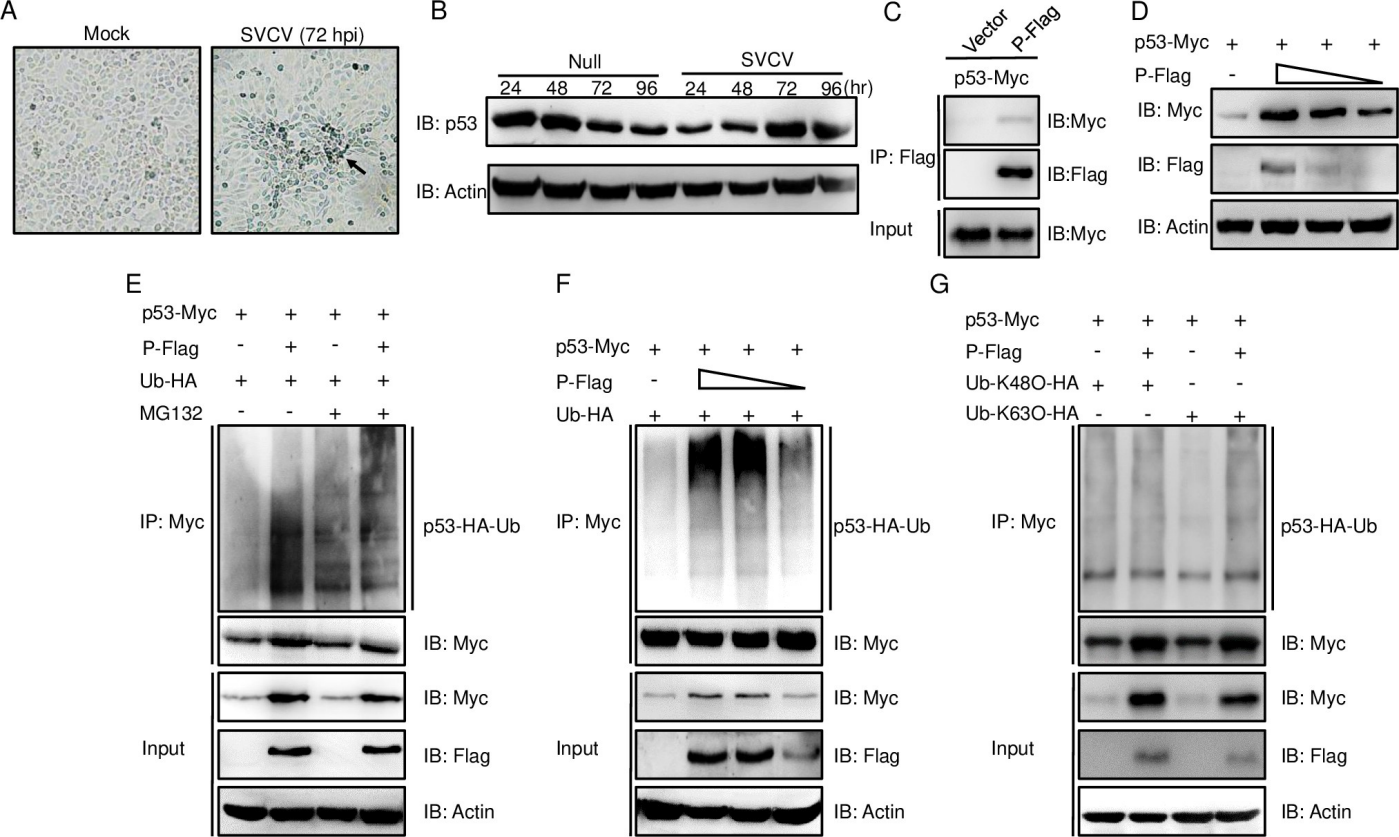
P protein of SVCV promotes K63-linked ubiquitination of p53. **(A)** SVCV infection induces cell apoptosis. ZFL cells were seeded in 6-well plates and uninfected (null) or infected with SVCV (MOI = 10) for 72 h, then the cells were subjected to microscopy analysis. **(B)** SVCV infection increases the expression of endogenous p53 protein at the late stage. ZFL cells were seeded in 6-well plates and uninfected (null) or infected with SVCV (MOI = 10) at various time points, and the WCL were detected by IB, with the anti-p53 and anti-β-actin Abs. **(C)** p53 associates with P protein in a mammalian overexpression system. HEK 293T cells seeded in 10 cm^2^ dishes were transfected with the indicated plasmids (5 μg each). After 24 h, cell lysates were IP with anti-Flag-affinity gels. Then the immunoprecipitates and WCL were analyzed by IB with the Abs indicated. **(D)** P protein increases the expression of p53 in a dose-dependent manner. EPC cells were seeded in 6-well plates and transfected with 1 μg p53-Myc and 1 μg empty vector, or P-Flag (1, 0.5, or 0.25 μg) for 24 h. Then the WCL were subjected to IB with the Abs indicated. **(E)** P protein promotes the ubiquitination of p53. EPC cells were seeded in 10 cm^2^ dishes and transfected with 5 μg p53-Myc, 5 μg P-Flag or empty vector, and 1 μg HA-Ub. At 18 h post-transfection, the cells were treated with DMSO or MG132 for 6 h. Cell lysates were IP with anti-Myc-affinity gels. Then the immunoprecipitates and WCL were analyzed by IB with the Abs indicated. **(F)** P protein positively regulates the ubiquitination of p53 in a dose-dependent manner. EPC cells were seeded in 10 cm^2^ dishes and transfected with 5 μg p53-Myc, 5 μg empty vector, or P-Flag (5, 2.5, or 1 μg), and 1 μg HA-Ub. At 18 h post-transfection, the cells were treated with MG132 for 6 h. Cell lysates were IP with anti-Myc-affinity gels. Then the immunoprecipitates and WCL were analyzed by IB with the Abs indicated. **(G)** P protein promotes K63-linked ubiquitination of p53. EPC cells were seeded in 10 cm^2^ dishes and transfected with 5 μg p53-Myc, 5 μg P-Flag or empty vector, and 1 μg Ub-K48O-HA or Ub-K63O-HA. At 18 h post-transfection, the cells were treated with MG132 for 6 h. Cell lysates were IP with anti-Myc-affinity gels. Then the immunoprecipitates and WCL were analyzed by IB with the Abs. All experiments were repeated for at least three times with similar results.

For the virus, host cell apoptosis is favorable for the assembled viron release, and p53 has been revealed as having a crucial role in the progress of apoptosis [31]. Therefore, we expect that cell apoptosis and p53 expression were regulated by SVCV at the late stage. Upon SVCV infection, cells underwent nuclear fragmentation and significant cell apoptotic bodies were observed at 72 hpi while the mock infection group was unaffected (Fig 5A). Then, the expression of p53 at the late stage of SVCV infection was monitored by IB. Intriguingly, consistent with the above results, although p53 was down-regulated for 24–48 hpi, it was up-regulated remarkably at 72–96 hpi (Fig 5B). With the subsequent co-transfection of P-Flag and p53-Myc, IB displayed that the anti-Flag Ab-immunoprecipitated protein complexes including P protein were also recognized by the anti-Myc Ab, which suggests that P protein interacted with p53 (Fig 5C). Moreover, a significant enhancement of p53 was improved by P protein dose-dependently (Fig 5D). Subsequently, the ubiquitination of p53 was assayed under treatment with MG132 and the ubiquitination of p53 was increased in the presence or absence of MG132 (Fig 5E). Furthermore, the ubiquitination of p53 was elevated by P protein dose-dependently (Fig 5F). Considering the different functions of K48-ubiquitination and K63-ubiquitination, Ub-K48O or Ub-K63O was co-expressed in the process of P protein regulated p53, and the K63-linked ubiquitination of p53 was notably increased when P protein was overexpressed (Fig 5G). Taken together, these data demonstrated that p53 was stabilized by K63-linked ubiquitination mediated by SVCV P protein.

### K358 is pivotal for p53 K63-linked ubiquitination to mediate both negative and positive regulation

**Fig 6:**
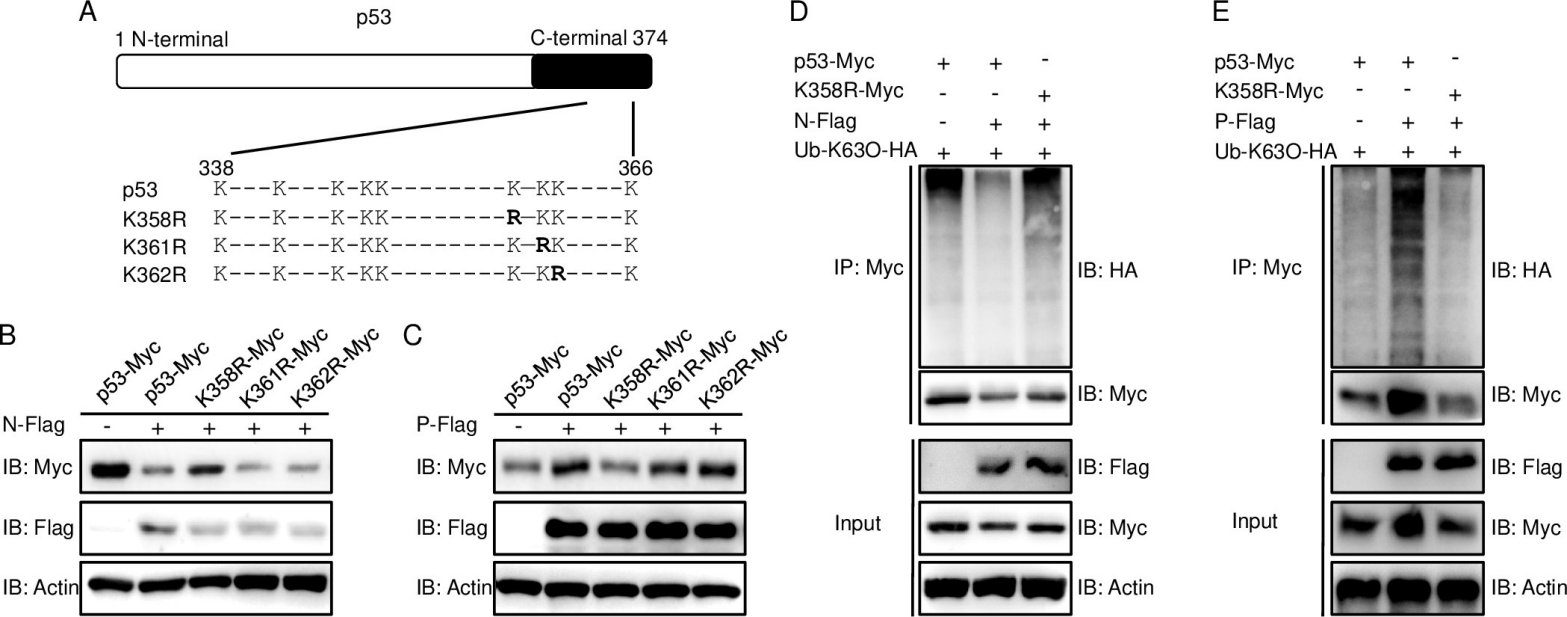
The K358 site is vital for K63-linked ubiquitination of p53. **(A)** A schematic of p53 site mutants. **(B and C)** The effect of N protein or P protein on the protein expression level of wild type and mutant p53. EPC cells were seeded in 6-well plates and transfected with 1 μg p53-Myc or p53 mutant together with 1 μg empty vector, or N-Flag (B), or P-Flag (C) for 24 h. Then the WCL were subjected to IB with the Abs indicated. **(D and E)** The effect of N protein or P protein on the K63-linked ubiquitination of wild type and K358R p53. EPC cells were seeded in 10 cm^2^ dishes and transfected with 5 μg p53-Myc or K358R-Myc, and 5 μg N-Flag (D) or P-Flag (E) or empty vector, and 1 μg Ub-K63O-HA. At 18 h post-transfection, the cells were treated with MG132 for 6 h. Cell lysates were IP with anti-Myc-affinity gels. Then the immunoprecipitates and WCL were analyzed by IB with the Abs. All experiments were repeated for at least three times with similar results.

In humans, the six lysines in the C-terminal of p53 are the major lysine residues that are ubiquitinated by E3 ligases [32]. Although there is little research about the ubiquitination site of zebrafish p53, zebrafish p53 is highly similar to mammalian p53 in both structure and function, and it has a 48% identical amino acid sequence to human p53 [33]. Therefore, according to the conserved amino acid sequence between human p53 and zebrafish p53 and professional ubiquitination site prediction (www.ubpred.org), the three lysines in the C-terminal of zebrafish p53 were mutated for ubiquitination site identification (Fig 6A). First, the stabilization of wild-type and mutant p53 were analyzed under the overexpression of N protein; the wild-type p53 was degraded compared to the control group, while the K358R of p53 was significantly rescued (Fig 6B). The results indicated that the K358 might be the ubiquitination site for N protein-mediated K63-linked ubiquitination. Meanwhile, K358R was remarkably maintained at a low expression level by stimulation with overexpressed P protein, which means that the K358 of p53 should be the functional site for P protein-mediated K63-linked ubiquitination (Fig 6C). Subsequently, to confirm the K63-linked ubiquitination of K358R p53, the immunoblot results during the overexpression of N protein demonstrated that the Ub-K63O of K358R p53 was higher than that of the wild-type p53 and nearly equaled to that of the empty vector group (Fig 6D). Next, the extent of the K63-linked ubiquitination of K358R p53 in the context of overexpressed P protein was monitored. The K63-linked ubiquitination of K358R p53 was lower than the wild-type p53 treated with the overexpression of P protein and approximated the empty vector group level, which demonstrated that the K358 of p53 was indispensable for P protein-mediated K63-linked ubiquitination (Fig 6E). These data suggested that the K358 of p53 was crucial for N protein- and P protein-mediated K63-linked ubiquitination.

## Discussion

The battles between aquatic viruses and fish or other lower vertebrates have remained mysterious to date because knowledge about the molecular mechanisms regarding viral invasion, viral infection, host immune responses, and so on is insufficient. This study reports an aquatic virus called SVCV that employs two distinct manners to regulate the host key factor p53 expression, lowering p53 with N protein and increasing p53 with P protein to promote viral infection. Interestingly, our previous studies showed that SVCV N protein degraded host MAVS to blunt IFN production, and P protein acted as a decoy of TBK1 interfering with IRF3 phosphorylation to abrogate IFN transcription [28, 29]. Combined with these studies, SVCV N and P proteins play multidimensional roles in controlling cell fate and antagonizing the IFN system. Actually, considering that they are viral non-structure proteins, SVCV N and P proteins should also participate in viral genome transcription and replication like in other rhabdoviruses [27]. This information indicates that two of the five viral proteins of SVCV possess multiple functions. That might be because, unlike DNA viruses, RNA viruses are usually composed of only a few proteins (e.g. SVCV only contains five proteins); hence, only efficient and multifunctional viral proteins are capable of accomplishing viral proliferation in host cells.

The major purpose of this study was to focus on p53 regulation by SVCV N and P proteins at protein level; only a minor space regarded the mRNA regulation of *p53* by N protein. In a viral infection, fish IFN is boosted to stimulate antiviral gene transcription [34]. Previous studies have shown that p53 mRNA is stimulated by type I IFN, which leads to the early upregulation of downstream target proteins to launch early apoptosis to inhibit viral replication [8, 35]. In our early study, the SVCV N protein degraded MAVS through the ubiquitin-proteasome pathway to block the host’s IFN production. Combined with these data, the downregulated *p53* mRNA by the overexpression of SVCV N protein in the current study might because that the expression of IFN was reduced. This mechanism should be one strategy of the virus to improve the infection. In addition, the inhibition of host target genes and even of the whole gene transcription level mediated by viral proteins is crucial for viral replication and proliferation [36–38]. The M protein of VHSV, which is an aquatic rhabdovirus, has been reported to decline the host transcription to escape the host immune response [39]. A similar function of SVCV M protein was observed in a series of preliminary experiments at our lab: the overexpression of M protein significantly inhibited the transcription level of both the endogenous genes and exogenous transfected genes. Since the promoters in the expression plasmids are strong ones, these data indicate that the transcriptional suppression mediated by rhabdovirus M protein targets RNA polymerase, but not specific host genes. Therefore, host transcription regulation plus the controlling of RNA polymerase should be crucial for viral invasion, in addition to protein-level modulation.

Upon virus infection, p53 is a pivotal regulator of cell-cycle arrest and cellular apoptosis, which are employed by host cells to defend against viruses. Thus, viruses possess evolutionary reasons to encode p53 antagonistic proteins. Usually, cell-cycle arrest is beneficial for viral early infection to plunder a host’s resources for viral replication, and late apoptosis is preferred for viron release. As examples, cell-cycle arrest, as mediated by p53, to delay apoptosis has been identified as a feasible mechanism to consequently enhance EMCV replication; regarding the regulation of apoptosis, adenovirus E1B-55K and E4-ORF6 proteins, human papillomavirus (HPV) E6, EBV EBNA-5, and KSHV vIRF4, as well as the vaccinia virus (VV) B1R kinase all induce the degradation of p53. Other proteins such as Adenovirus E1A and HPV E7 maintain p53 stability [40, 41]. The strategies utilized by viruses to modulate cellular p53 to affect host apoptosis should finally promote viral replication and proliferation. In this study, the SVCV N protein induces cell-cycle arrest, and the P protein facilitates apoptosis by stabilizing p53, which indicates the strategies that might be employed by a virus at different time points of infection.

## Materials and methods

### Cells, viruses

Human embryonic kidney (HEK) 293T cells were provided by Dr. Xing Liu (Institute of Hydrobiology, Chinese Academy of Sciences) and were grown at 37°C in 5% CO_2_ in Dulbecco’s modified Eagle’s medium (DMEM; Invitrogen) supplemented with 10% fetal bovine serum (FBS, Invitrogen). Zebrafish liver (ZFL) cells (American Type Culture Collection, ATCC) were cultured at 28°C in 5% CO_2_ in Ham’s F12 nutrient mixture medium (Invitrogen) supplemented with 10% FBS. p53 stably-expressed ZFL cells were transfected with GFP or GFP-p53 and were subjected to G418 selection to enrich GFP positive cells. Epithelioma papulosum cyprini (EPC) cells were obtained from China Center for Type Culture Collection (CCTCC) and were maintained at 28°C in 5% CO_2_ in medium 199 (Invitrogen) supplemented with 10% FBS. Spring viremia of carp virus (SVCV) was propagated in EPC cells until cytopathic effect (CPE) was observed, then the culture medium with cells was harvested and stored at -80°C until needed.

### Cell cycle analysis

The ratio of cells in each phase of the cell cycle was determined by DNA content using propidium iodide (PI) staining followed by flow cytometric analysis. The cells plated at a density of 1 × 10^6^ cells/flask were treated with the indicated multiplicity of infection (MOI) of SVCV for the indicated times as described in the figure legends. The cells were trypsinized, washed twice with PBS, and fixed with 70% ice-cold ethanol at −20°C overnight. Fixed cells were washed with cold PBS and resuspended with PI staining solution containing 50 μg/mL PI (Sigma-Aldrich), 100 μg/mL RNase A (TIANGEN Biotech), 0.02% Triton X-100, and incubated in the dark for 30 min. The samples were analyzed using a flow cytometer (BD Biosciences).

### Plasmid construction and reagents

The open reading frame (ORF) of zebrafish p53 (GenBank accession number NM_001271820.1) and p53 mutants was generated by PCR and then cloned into pCMV-Myc (Clontech). The expression plasmids for Flag-tagged N, and Flag-tagged P were described previously [42, 43]. All constructs were confirmed by DNA sequencing. MG132 was purchased from Sigma-Aldrich and used at a final concentration of 20 μM/ml.

### Transient transfection and virus infection

Transient transfections were performed in EPC cells seeded in 6-well or 24-well plates or ZFL cells seeded in 6-well plates by using X-tremeGENE HP DNA Transfection Reagent (Roche) according to the manufacturer’s protocol. For the antiviral assay using 24-well plates, EPC cells were transfected with 0.5 μg Myc-N or the empty vector. At 24 h post-transfection, cells were infected with SVCV (MOI = 10). After 2 or 3 d, supernatant aliquots were harvested for detection of virus titers, the cell monolayers were fixed by 4% paraformaldehyde (PFA) and stained with 1% crystal violet for visualizing CPE. For virus titration, 200 μl of culture medium were collected at 48 h post-infection and used for plaque assay. The supernatants were subjected to 4-fold serial dilutions and then added (100 μl) onto a monolayer of EPC cells cultured in a 96-well plate. After 48 or 72 h, the medium was removed, and the cells were washed with PBS, fixed by 4% PFA and stained with 1% crystal violet. Results are representative of three independent experiments.

### RNA interference (RNAi) experiment

EPC cells were seeded into 6-well plates overnight and transfected with 100 nM siRNA of N or the negative control (siNC by using the X-tremeGENE HP DNA transfection reagent (Roche) according to the manufacturer’s protocols. siRNA of N and siCon were obtained from GenePharma (Shanghai, China). The following sequences were targeted for SVCV N: siN#1 (GCAUGUGAAUGCUUAUCUATT), siN#2 (GCCAAAUCACCAUACUCAATT), and siN#3 (GCCUCGCGAUAACUCAGUUTT).

### RNA extraction, reverse transcription, and qPCR

Total RNA was extracted by the TRIzol reagent (Invitrogen). First-strand cDNA was synthesized by using a GoScript reverse transcription system (Promega) according to the manufacturer’s instructions. qPCR was performed with Fast SYBR green PCR master mix (Bio-Rad) on the CFX96 real-time system (Bio-Rad). PCR conditions were as follows: 95°C for 5 min and then 40 cycles of 95°C for 20 s, 60°C for 20 s, and 72°C for 20 s. *β-actin* gene was used as an internal control. The relative fold changes were calculated by comparison to the corresponding controls using the 2^-ΔΔCt^ method. Three independent experiments were conducted for statistical analysis. The following gene-specific primer sequences were utilized for the qPCR: *Dr*p53, 5’-CCCGGATGGAGATAACTTG-3’ and 5’-CACAGTTGTCCATTCAGCAC-3’; *Dr*p21, 5’-GACCAACATCACAGATTTCTAC-3’ and 5’-TGTCAATAACGCTGCTACG-3’; *Dr*CyclinG1, 5’-CATCTCTAAAAGAGGCTCTAG-3’ and 5’-CACACAAACCAGGTCTCCAG-3’; *Dr*β-actin, 5’-CACTGTGCCCATCTACGAG-3’ and 5’-CCATCTCCTGCTCGAAGTC-3’; SVCV-N, 5’-CCTACAACAGCCGCAGAGAC-3’ and 5’-GCACTCAACCACAGCATCCA-3’.

### Co-immunoprecipitation (Co-IP) assay

The HEK 293T cells or EPC cells seeded into 10 cm^2^ dishes overnight were transfected with a total of 10 μg of the plasmids indicated on the figures. At 24 h post-transfection, the medium was removed carefully, and the cell monolayer was washed twice with 10 ml ice-cold PBS. Then the cells were lysed in 1 ml of radioimmunoprecipitation (RIPA) lysis buffer [1% NP-40, 50 mM Tris-HCl, pH 7.5, 150 mM NaCl, 1 mM EDTA, 1 mM NaF, 1 mM sodium orthovanadate (Na_3_VO_4_), 1 mM phenyl-methylsulfonyl fluoride (PMSF), 0.25% sodium deoxycholate] containing protease inhibitor cocktail (Sigma-Aldrich) at 4°C for 1 h on a rocker platform. The cellular debris was removed by centrifugation at 12,000 × *g* for 15 min at 4°C. The supernatant was transferred to a fresh tube and incubated with 30 μl anti-Flag affinity gel or anti-Myc affinity gel (Sigma-Aldrich) overnight at 4°C with constant agitation. These samples were further analyzed by immunoblot (IB). Immunoprecipitated (IP) proteins were collected by centrifugation at 5000 × *g* for 1 min at 4°C, washed three times with lysis buffer and resuspended in 50 μl 2 × SDS sample buffer. The immunoprecipitates and whole cell lysates were analyzed by IB with the indicated antibodies (Abs).

### Recombinant protein purification and polyclonal antibody development

For prokaryotic expression, the ORF of N or P protein was cloned into the *EcoR* I and *Xho* I sites of pET28a(+) vector. Prokaryotic expression plasmids were transformed into *Escherichia coli* BL21 (DE3)-CodonPlus-RIL competent cells (Stratagene). Recombinant proteins were expressed by induction with isopropyl-β-D-thiogalactopyranoside (IPTG) and purified from the supernatants of cell lysates under native condition. The His-tagged recombinant N or P was bound to a Ni-NTA Superflow resin (Qiagen) by rocking at 4°C overnight. The resin was in turn washed with wash buffer 1 (50 mM NaH_2_PO_4_, 300 mM NaCl, pH 8.0; containing 20 mM imidazole), wash buffer 2 (containing 40 mM imidazole), and wash buffer 3 (containing 60 mM imidazole). The affinity-purified proteins were further purified by gel filtration using a Superdex 75 column on an AKTA FPLC system (GE Healthcare). Purity and concentration of the purified recombinant proteins were determined by SDS-PAGE and BCA Protein Assay (Thermo Scientific). Recombinant proteins were applied to immunize white rabbits to raise polyclonal antibodies against SVCV N or P protein, respectively.

### Immunoblot analysis

Immunoprecipitates or whole cell lysates were separated by 10% SDS-PAGE and transferred to polyvinylidene difluoride (PVDF) membrane (Bio-Rad). The membranes were blocked for 1 h at room temperature in TBST buffer (25 mM Tris-HCl, 150 mM NaCl, 0.1% Tween 20, pH 7.5) containing 5% nonfat dry milk, probed with the indicated primary Abs at an appropriate dilution overnight at 4°C, washed three times with TBST, and then incubated with secondary Abs for 1 h at room temperature. After three additional washes with TBST, the membranes were stained with the Immobilon Western chemiluminescent horseradish peroxidase (HRP) substrate (Millipore) and detected by using an ImageQuant LAS 4000 system (GE Healthcare). Abs were diluted as follows: anti-β-actin (Cell Signaling Technology) at 1:1,000, anti-Flag/HA (Sigma-Aldrich) at 1:3,000, anti-Myc (Santa Cruz Biotechnology) at 1:2,000, and HRP-conjugated anti-mouse IgG or anti-rabbit IgG (Thermo Scientific) at 1:5,000. Results are representative of three independent experiments.

### Statistics analysis

Data are expressed as mean ± standard deviations (SDs) which obtained by measuring each sample in triplicate. The *p* values were calculated by one-way analysis of variance (ANOVA) with Dunnett’s post hoc test (SPSS Statistics, version 19; IBM). A *p* value < 0.05 was considered statistically significant.
